# Complex networks of marine heatwaves reveal abrupt transitions in the global ocean

**DOI:** 10.1038/s41598-021-81369-3

**Published:** 2021-01-18

**Authors:** Lisandro Benedetti-Cecchi

**Affiliations:** 1 Department of Biology, University of Pisa, Via Derna 1, 56126 Pisa, Italy; 2 Stazione Zoologica Anton Dohrn, 80121 Naples, Italy; 3 CoNISMa, Piazzale Flaminio 9, 00196 Rome, Italy

**Keywords:** Climate sciences, Ecology, Ocean sciences

## Abstract

Understanding how marine heatwaves (MHWs) unfold in space and time under anthropogenic climate change is key to anticipate future impacts on ecosystems and society. Yet, our knowledge of the spatiotemporal dynamics of MHWs is very limited. Here, I combine network theory with topological data analysis and event synchronization to high-resolution satellite data and to a set of Earth System Model simulations to reveal the dynamical organization of complex MHW networks. The analysis reveals that MHWs have already crossed a tipping point separating highly synchronized preindustrial MHWs from the more extreme, but less coherent warming events we experience today. This loose spatiotemporal organization persists under a reduced RCP 2.6 emission scenario, whereas a second abrupt transition towards a permanent state of highly synchronized MHWs is foreseen by 2075 under a business-as-usual RCP 8.5 scenario. These results highlight the risks of abrupt ocean transitions, which may dramatically affect marine life and humanity by eroding valuable time for adaptation to climate change.

## Introduction

There is increasing evidence that MHWs, persistent positive anomalies in sea surface temperatures (SSTs), can occur everywhere at any time in the ocean under global warming^1–3^. A surge of warm anomalies in SSTs have been recorded in the last two decades, with record-breaking events causing dramatic impacts to marine ecosystems, economies and society worldwide^4–16^. Observed trends of increasing intensity, frequency, duration and spatial extent of MHWs are projected to endure throughout the twenty-first century^1,13,17^. Key insights into the dynamics of MHWs have emerged from consideration of events at individual locations (cells, pixels) and through a global synthesis of their collective characteristics. However, studies have typically examined temporal and spatial patterns separately, often aggregating data in one or the other dimension to disclose the relevant trends^3,13,17^. Here, I use the framework of complex networks^18–21^ to reveal the global dynamical organization of MHWs, which cannot be captured by treating space and time separately. A network representation of MHWs is appealing because it allows to: (1) explore the full spatiotemporal continuum of SST data; (2) uncover nonlinearities, thresholds and the relevant scales of change that may be masked when aggregating data in space and time and (3) employ powerful statistical tools of network analysis. When applied to observed and simulated time series of SSTs, this approach reveals previously unrecognized signatures of potential devastating abrupt transitions (tipping points) in the global ocean.

**Figure 1:**
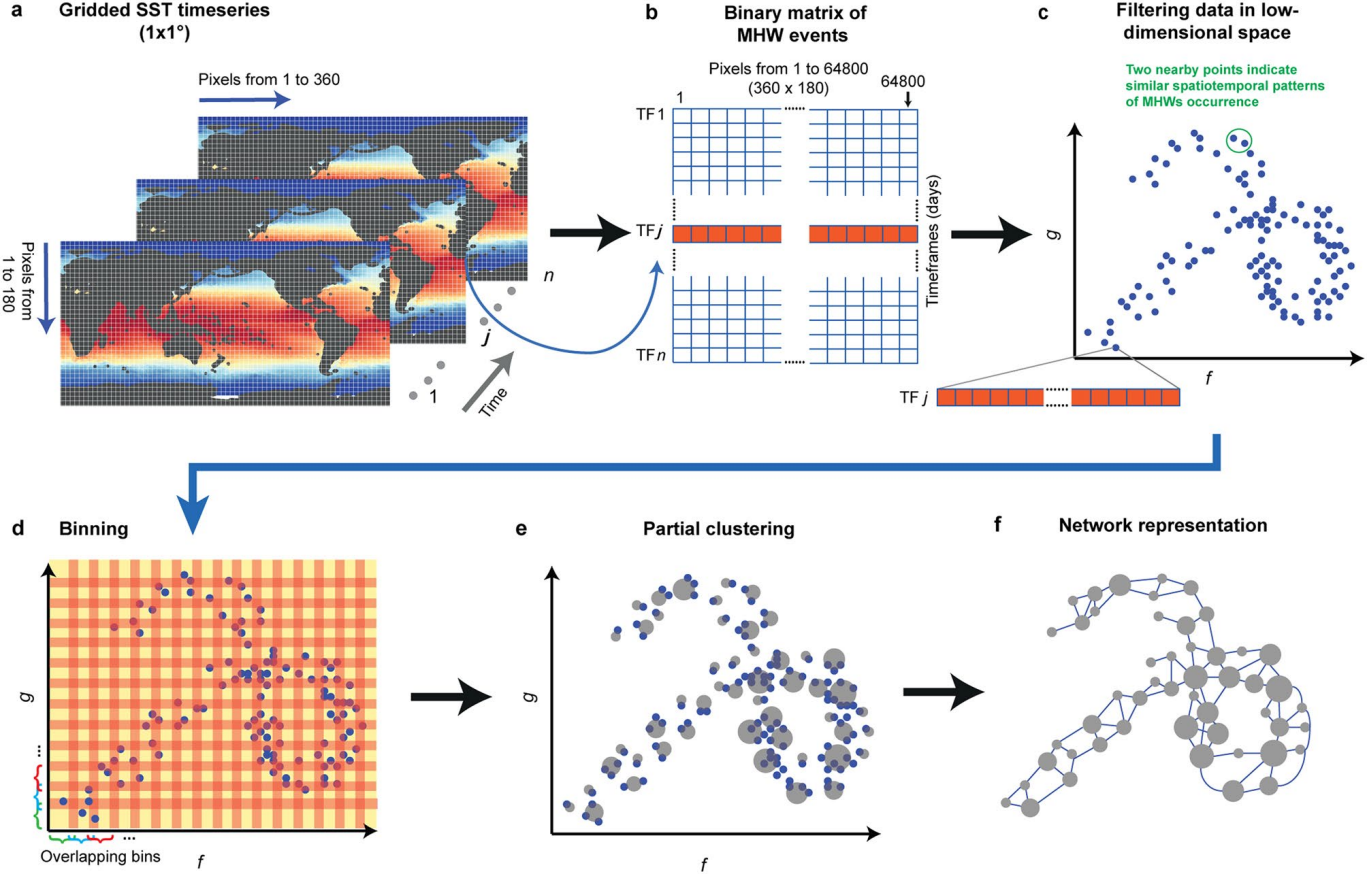
Workflow to reveal the dynamical organization of marine heatwaves (MHWs) using Mapper. (**a**) Raster stacks of daily sea-surface-temperature (SST) timeseries at 1° × 1° resolution. There is one raster stack for observed SSTs and one for each of the simulated emission scenarios (Historical, RCP 2.6 and RCP 8.5). MHWs are identified and characterized from SST timeseries for each pixel in each raster stack using the definition proposed by Hobday et al.^27^. Maps were produced in R 4.0.2^29^ (https://www.R-project.org/). (**b**) Each raster stack is collapsed into a binary 2D matrix where rows are timeframes (TF) and columns are 1° × 1° cells arranged sequentially to represent the occurrence of MHWs across the global ocean. Data entries in the matrix indicate the absence (zero) or the occurrence (one) of an MHW for a given day and pixel. (**c**) Filtering step or non-linear dimensionality reduction, where MHW occurrence data is projected into a lower two-dimensional space (represented by dimensions *g* and *f*). (**d**) Binning step where the low-dimensional space is divided into overlapping bins whose size is determined by the resolution parameter *R* and degree of overlap is determined by the gain parameter *G*. (**e**) Partial clustering of timeframes within nodes to obtain a compressed representation of the original data. Each node contains from one to several timeframes with similar spatial patterns of MHW occurrence. The size of each node reflects the number of timeframes included in the cluster. (**f**) Final representation of an MHW network. Nodes that share at least one timeframe are connected through an edge. Note that because bins overlap to a certain degree, the Mapper algorithm involves oversampling of the original data.

To represent MHWs as networks without collapsing data in space or time, I used the Mapper algorithm from the field of Topological Data Analysis (TDA)^22–24^. TDA is an emerging and reliable technique to identify patters and shapes in spatiotemporal data that has been successfully applied to analyze complex dynamical systems including brain activity maps^22^, cancer disease^25^ and financial markets^26^. To assess the properties of past and future MHWs networks, I applied the standardized definition of a marine heatwave proposed by Hobday et al.^27^ to (i) remotely sensed daily global SST data over the period 1982–2018^4,28^ and (ii) daily output from 12 fully coupled Earth System Models covering the period 1861–2100. To apply the TDA-based Mapper to each dataset, MHW events were converted to a binary matrix of zeros and ones (indicating the absence or presence of a MHW, respectively), with each row of the matrix representing the entire globe for a given day (hereafter timeframe). The Mapper algorithm is then employed on the binary matrix to perform four steps: filtering, binning, partial clustering and network representation (Fig. 1). Each node in the network is a cluster of one to several timeframes and nodes are linked together if they share at least one timeframe. Timeframes within linked nodes have similar spatial patterns of MHW occurrences.

The full network encodes both spatial and temporal information of MHW events in a low-dimensional compressed representation produced by the clustering of timeframes within nodes. This approach allows for great flexibility to explore spatiotemporal dynamics and transitions^22^ and complements methodologies used to construct functional climate networks where nodes correspond to individual spatial locations^18^. Because nodes encode both spatial and temporal information of MHW occurrences, prominent events can be extracted from TDA-based networks and their characteristics (e.g., duration, spatial extent) can be visualized as maps and time series (Supplementary Fig. 1). To navigate through the networks and to interactively explore their spatial and temporal properties, I developed a web tool that is available at: calcoloecologia.biologia.unipi.it:3838/MHW_App) based on R 4.0.2^29^.

## Results

### Network representation of MHWs

**Figure 2:**
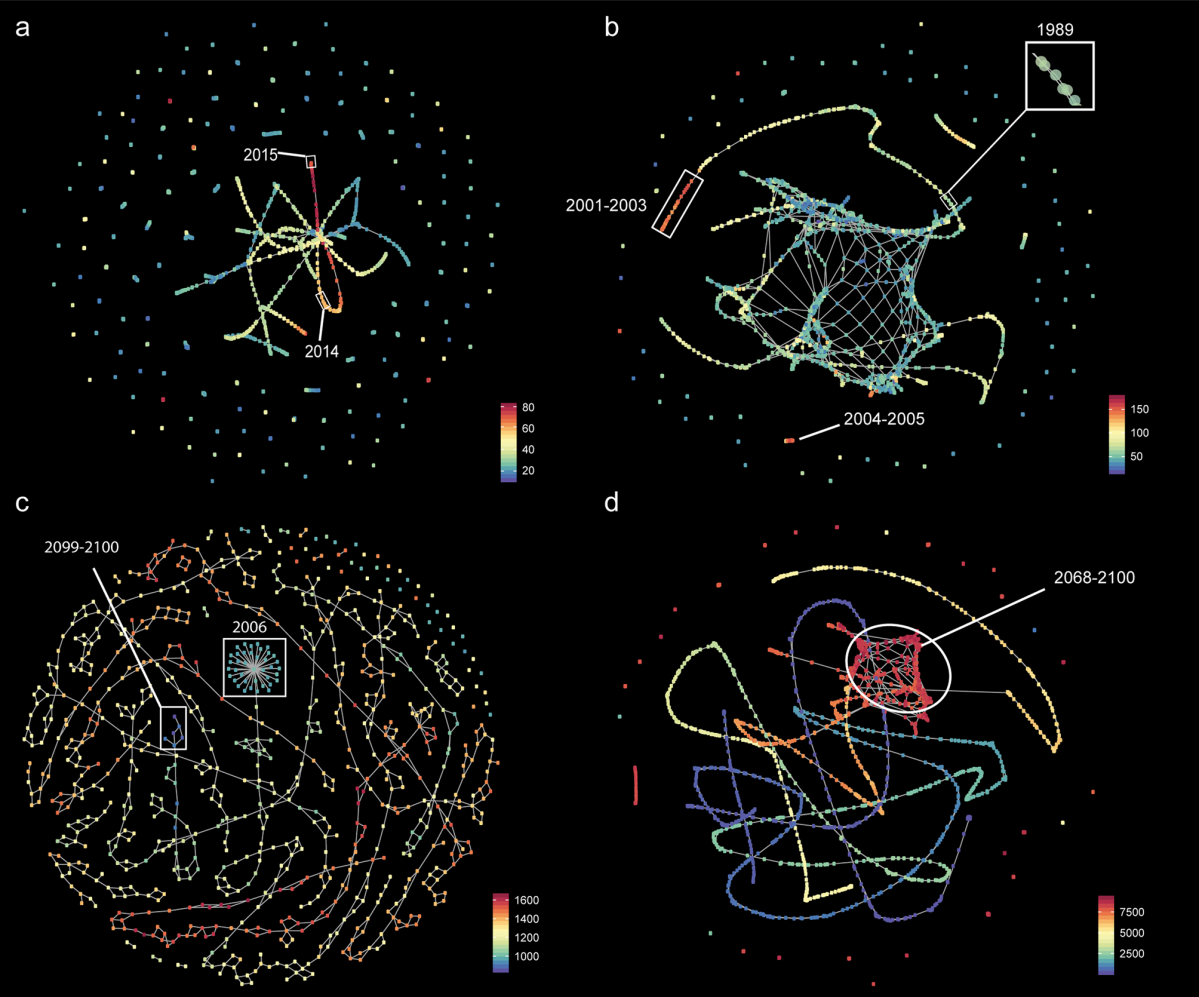
A network representation of globally distributed MHWs. Networks generated by the TDA-based Mapper algorithm are annotated by the duration of MHWs averaged across timeframes in each node. (**a**) Network of observed SSTs (1981–2018) showing the prevalence of isolated events. The white frames highlight subsets of nodes encoding part of the long-lasting northeastern Pacific MHW known as ‘the blob’ (see also Supplementary Fig. 1a); (**b**) Network of simulated SSTs from the Historical CO_2_ emission scenario (1861–2005). The inset shows the ramification of loosely connected nodes starting in 1989 and initiating a pathway of increasing MHW duration toward the end of the historical period (white frame and isolated 2004–2005 nodes); (**c**) Network of simulated SSTs from the RCP 2.6 CO_2_ emission scenario (2006–2100). There is no evidence of increasing duration of MHWs from the beginning to the end of the simulation period (white frames); (**d**) Network of simulated SSTs from the RCP 8.5 CO_2_ emission scenario (2006–2100). A region of densely connected nodes emerges in the second half of the simulation period (red nodes in the white circle), corresponding to persistent MHWs covering vast areas of the global ocean (Supplementary Fig. 1g,h).

**Figure 3:**
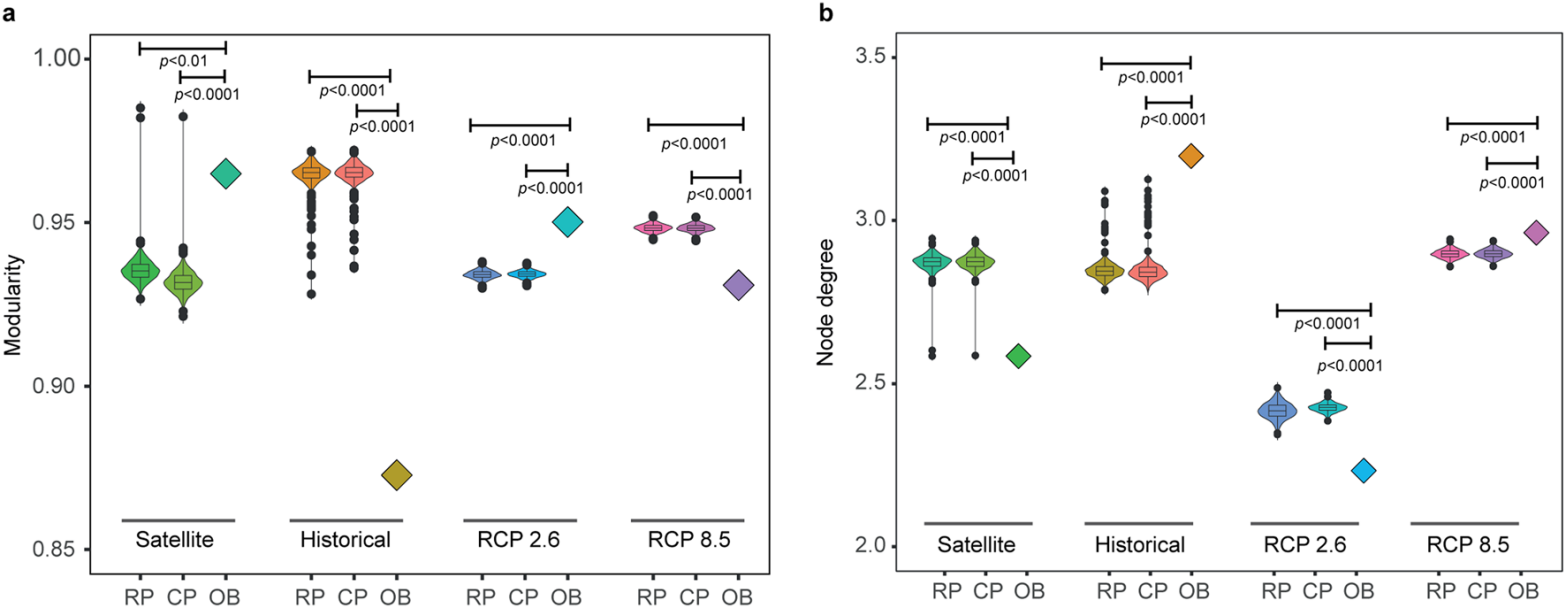
Statistical analysis of networks. (**a**) Modularity; (**b**) Node degree. Panels show violin and box plots derived from null models under random and constant phase randomization (RP and CP, respectively; *n* = 1000) and observed (OB) statistics for remotely sensed SSTs (satellite) and for each of the Historical, RCP 2.6 and RCP 8.5 emission scenarios considered in this study. Violin plots show kernel density estimates at different values of the data; box-plots show the median, an upper and lower hinge corresponding to the 25th and 75th percentiles of the distribution (first and third quartiles) and the whiskers, which show data dispersion up to 1.5 times the inter-quantile range; points are outliers.

The MHW networks of observed and simulated SST timeseries have different topological characteristics (Fig. 2). The network of observed MHWs (1981–2018) has a significant modular structure (nodes organized in clusters) (Fig. 3a), with many groups of one or few nodes and a core region that includes the long-lasting 2014–2015 northeastern Pacific MHW^5^ (Fig. 2a, Supplementary Fig. 1). Node degree (the number of links incident to a node) is significantly lower than in null models, reflecting the prevalence of isolated nodes (Fig. 3b). The network obtained from simulated SSTs under the historical CO_2_ emission scenario (1861–2005) has a set of central and densely connected nodes and ramifications of loosely connected nodes (Fig. 2b). This topological structure has significantly lower modularity and significantly larger node degree compared to null models (Fig. 3). The network shows longer and more intense MHWs towards the end of the simulation period (Fig. 2b, Supplementary Figs. 1, 2). The network originating from the RCP 2.6 scenario (2006–2100) has loosely connected nodes (Fig. 2c) with significantly larger modularity and significantly lower node degree compared to null models, a pattern similar to that identified in the network of observed MHWs (Fig. 3). Ground-breaking events are distributed across the network with no evidence of worsening conditions towards the end of the century (Fig. 2c, Supplementary Figs. 1, 2). The network originating from the RCP 8.5 scenario (2006–2100) shows ramifications of loosely connected nodes and a region of densely connected nodes that reflects record-breaking MHWs for duration and intensity that emerge in the second half of the twenty-first century (Fig. 2d, Supplementary Figs. 1, 2). Network modularity is significantly lower and node degree significantly larger than in null models (Fig. 3).

### Transitions between dynamical regimes

**Figure 4:**
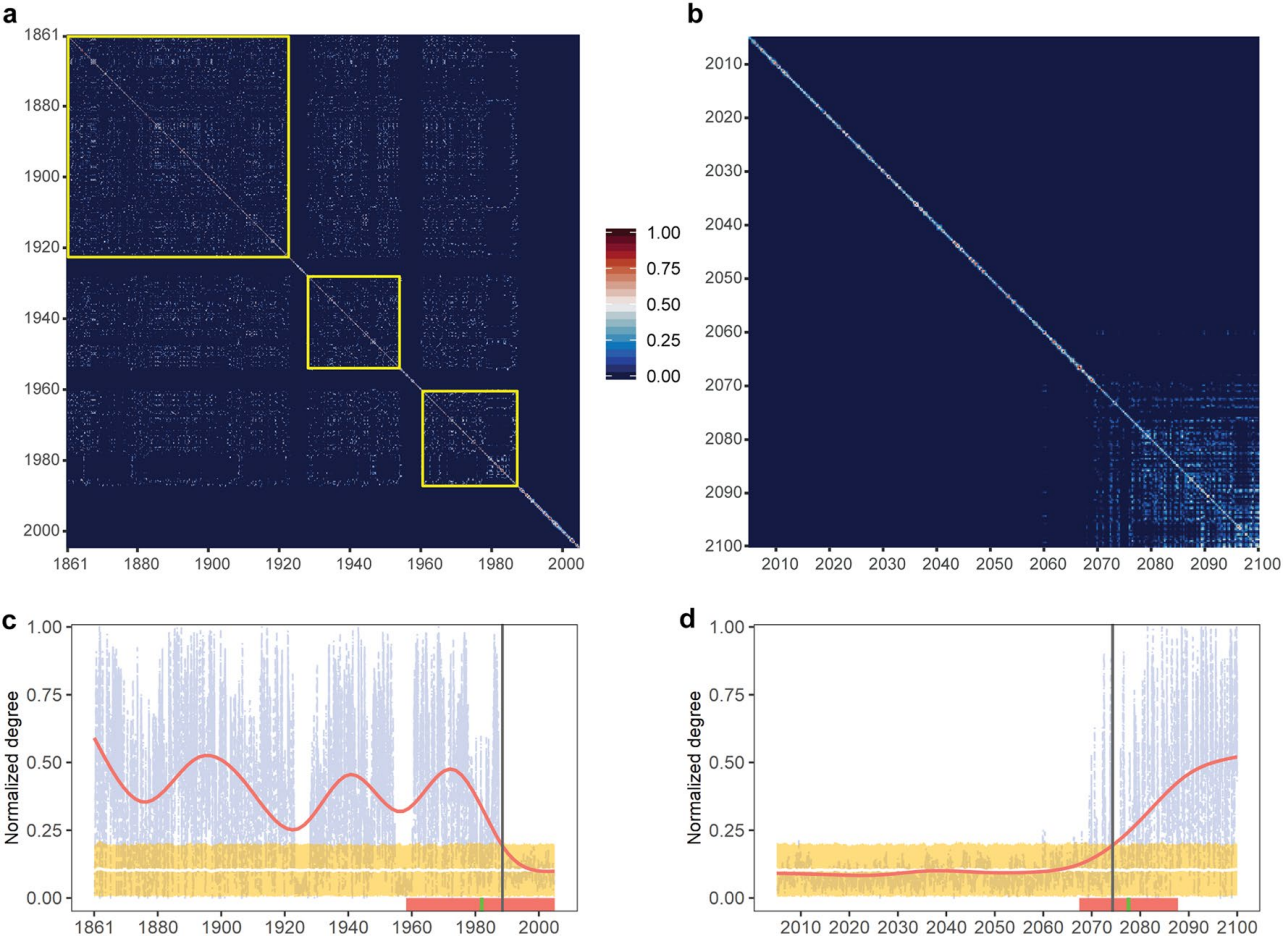
Temporal transitions of global MHW dynamics. Panels show temporal connectivity matrices (TCMs) originating from (**a**) historical and (**b**) RCP 8.5 networks. A TCM shows the similarity of each timeframe with all other timeframes. Yellow frames in (**a**) highlight the timescales of recurrent MHWs. Temporal transitions are captured by the normalized node degree of the TCMs for (**c**) historical and (**d**) RCP 8.5 scenarios. Red lines in (**c**,**d**) are Generalized Additive Model (GAM) fits to node degree data; yellow bands are 95% confidence intervals of random phase null models (*n* = 1000, with average trend in white); vertical gray lines show years of transition determined from the intersection of GAM fits with the upper confidence limit of null models; vertical green lines and orange bands show the median transition year estimated from individual Earth System Models (*n* = 12, Supplementary Fig. 3) and the bootstrap error for the median, respectively.

The co-occurrence of regions with high and low node degree within the historical and RCP 8.5 networks suggests transitions between distinct dynamical regimes in the evolution of MHWs. High node degree implies recurrent patterns over large time scales, whereas low node degree indicates short-term similarity. To better characterize the dynamical regimes of MHWs and to determine the time scales involved, I converted each network into a temporal connectivity matrix (TCM)—i.e. an adjacency matrix in the temporal domain^22^. A TCM represents connectivity (similarity) among timeframes, which are considered connected if they belong to connected nodes in the network or if they share the same node. Connectivity in the TCM means that similar configurations of MHWs appear at different times. The TCM derived from the historical network has a modular structure with three periods of persistent connectivity alternating with three periods of short-term similarity (Fig. 4a). A standard change point detection algorithm (see “Methods”) allows to precisely determine the duration of the different periods. The longest period of connected timeframes lasts 63 years, from 1861 to 1923, whereas the two other periods of connectivity last 26 (1929–1955) and 27 (1961–1988) years, respectively (yellow frames in Fig. 4a). In contrast, connectivity ranges from one day to two years during periods of low similarity (voids) in the TCM.

These patterns of connectivity separate two distinct regimes in the evolution of MHWs: one characterized by recurrent patterns over decadal scales and one in which temporal similarity decays over time scales of days to years. The normalized node degree of the TCM (the total number of connections for each timeframe) alternates between persistent periods of significant connectivity to periods of loss of connectivity, capturing transitions between regimes (Fig. 4c). These shifts are abrupt, as they occur over very short time scales compared to the duration of the following (or preceding) period of high or low connectivity and correspond to the alternating patterns between high and low (voids) of similarity observed in the TCM (Fig. 4a). A Generalized Additive Model (GAM) smooth function fitted to the normalized node degree data identifies a significant collapse of connectivity by 1989 (determined from the intersection of the smooth function with the upper confidence limit of a null model, see “Methods”), a pattern that persists until the end of the simulation period (Fig. 4c). A similar result is obtained using data from individual Earth System Models, which identify 1982 as the median year of collapse (Supplementary Fig. 3). However, there is considerable variation in the outcome of individual models, which adds uncertainty to this estimate (± 24 years, bootstrap standard error) (Fig. 4c, Supplementary Fig. 3).

Remarkably, short-term connectivity characterizes the network of observed MHWs and endures throughout the twenty-first century under the RCP 2.6 emission scenario (Supplementary Fig. 4). In contrast, a second transition back to large and significant temporal scales of connectivity is observed under the RCP 8.5 emission scenario, with node degree increasing again by 2068 and remaining above the upper confidence limit of the random phase null model from 2075 until the end of the century (Fig. 4b,d). A similar result is obtained using data from individual Earth System Models, which also identify 2078 (± 10 years, median and bootstrap standard error, *n* = 12) as the year of transition towards significant temporal scales of connectivity (Supplementary Fig. 3). A sensitivity analysis shows that network properties and patterns of connectivity are robust to a wide range of parameter perturbations in the TDA-based Mapper algorithm (see “Methods” and Supplementary Figs. 5–8).

### Synchronization of MHWs

Transitions between periods of different temporal connectivity may also involve changes in the spatial scales at which MHWs organize. I use event synchronization to determine the spatial scales of MHWs associated with periods of high or low temporal connectivity. This analysis compares the timeseries for all possible combinations of the 1° × 1° grid cells and determined the global distribution of spatial distances of significant connections within different time windows of a TCM. As shown in a previous study, event synchronization can reveal abrupt shifts in the relevant scales of synchronization of extreme events and inform on the underlying dominant processes, using the distribution of all possible great-circle distances on the Earth’s surface as a benchmark^18^. Positive (negative) deviations from the benchmark highlight probabilities of synchronization of MHWs above (below) what can be expected from geographic distance alone.

**Figure 5:**
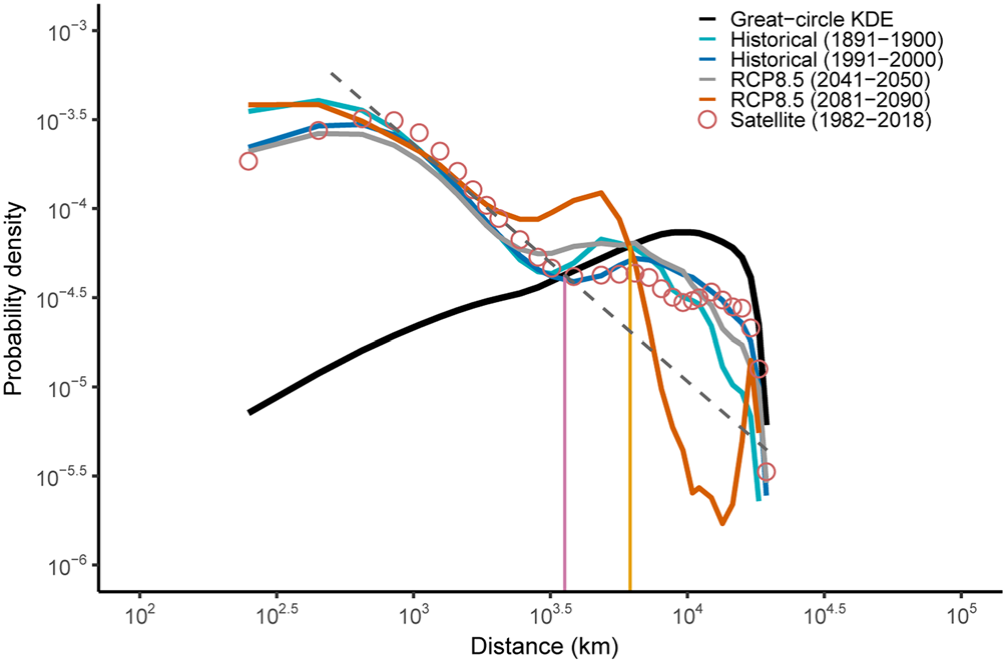
Distance distribution of MHW synchronization. Probability density function of spatial distances of significant event synchronization for different time windows within the historical and RCP8.5 scenarios and for the whole period of observed (satellite) data. The black line shows the kernel density estimates (KDE) of the distribution of all possible great-circle distances of MHWs. The dashed line is the power-law fit to the satellite distribution within the range of 500–3600 km (power-law exponent *α* = 1.33, *R*^2^ = 0.96). A nearly identical power-law fit in the same spatial range is obtained for historical distributions in the period 1991–2000 (power-law exponent *α* = 1.32, *R*^2^ = 0.97, fit not shown). The pink vertical line at about 3600 km marks a shift from regional to large-scale patterns of MHW synchronization for satellite and historical (1991–2000) distance distributions. The yellow vertical line shows extended spatial scales of synchronization at about 6000 km for the thick-tailed distributions of historical (1891–1900) and RCP 8.5 (both periods) distances. At distances below 400 km the distribution is biased by the resolution of grid cells (1° × 1°), with smaller geographical distances near the poles.

The distributions of spatial distances computed within a time window of 10 years during the most recent period of low temporal connectivity in the historical TCM (1989–2000, Fig. 5) and for the entire timeseries of contemporary satellite observations (1982–2018) decay as power-laws ($p \propto d^{ - \alpha }$) with very similar power exponents (α = 1.321, *R*^2^ = 0.98 and α = 1.329, *R*^2^ = 0.96, respectively) for distances below about 3600 km. Beyond that distance, the distributions become thick-tailed and large-scale synchronizations are more likely than expected from power-law scaling (Fig. 5). Thick-tail distributions are particularly pronounced in the range of distances of 3600–6000 km for the spatial distances obtained during periods of high connectivity (within time windows of 10 years) in the historical TCM (1891–1900) and for both periods of low (2041–2050) and high (2081–2090) connectivity in the RCP 8.5 TCM (Fig. 5). Larger connectivity in the RCP 8.5 TCM corresponds to larger probabilities of synchronization of MHWs at scales between 3600 and 6000 km. Repeating the analysis for different time windows supports the decline of large-scale synchronization from the first decades of the historical period (1861–1923) to the present, with thick-tailed regions raising again towards the end of the twenty-first century under the RCP 8.5 scenario (Supplementary Fig. 9). All distributions eventually converge to the distribution of all possible great-circle distances (Fig. 5).

## Discussion

I have used a novel approach based on TDA and the Mapper algorithm to implement a network analysis of observed and simulated MHWs under Historical, RCP 2.6 and RCP 8.5 emission scenarios. This approach in combination with event synchronization analysis has identified distinct spatiotemporal regimes in the dynamics of MHWs, with characteristic patterns of network structure, time scales of connectivity and spatial scales of synchronization, offering an unprecedented view of the dynamical organization of MHWs. The historical network shows long periods of temporal similarity of MHWs during preindustrial times. This pattern lasts until the late 1980s, when network connectivity drops dramatically marking a transition towards shorter temporal scales of similarity. This condition of low connectivity characterizes the network of observed MHWs and persists throughout the simulation period in the RCP 2.6 network. In contrast, a second abrupt transition back to significant temporal connectivity of MHWs occurs by the second half of the twenty-first century under the RCP 8.5 emission scenario. In addition to providing novel insights into the dynamical organization of MHWs, networks also encode the trends of increasing duration, intensity, frequency and spatial extent of MHWs identified in recent studies under current and future global warming scenarios^1–3^.

MHWs result from physical processes operating at different scales. At local to regional scales (10s–100s of km), mixed-layer processes such as air-sea heat fluxes, ocean-heat advection and vertical mixing of heat are considered direct drivers of MHWs^2,30^. These influences are modulated by mesoscale climate modes (e.g., El Niño Southern Oscillation, the Indian Ocean Dipole) operating at scales of 1000s of km, and their teleconnections at further larger scales (e.g., through Rossby waves)^2,30^. The distance distributions of significantly synchronized MHWs capture these scale-dependent processes. The power-law scaling observed for contemporary and historical MHWs during time windows of low connectivity likely reflects the influence of local- to regional-scale processes. The break point observed at 3600 km demarcates the transition to a domain where the probability of large-scale synchronization is much larger than expected from the distance-decaying patterns, which can be interpreted as a signature of meso- to large-scale influences.

These results are remarkably similar to what previously described for rainfall teleconnections, where a break-point in the power-law scaling occurred at distances of about 2500 km, beyond which the distribution of significant connections matched that generated by all possible great-circle distances^18^. These similarities reflect strong ocean–atmosphere coupling and suggest general principles underlying the spatiotemporal organization of climate extremes in the ocean and the atmosphere. The strengthening effect of tropical teleconnections on the atmospheric forcing that led to the 2015 record-breaking MHW in the northeastern Pacific is a case in point^30^. The larger spatial scale at which a thick-tail region emerges in the distance distribution of MHWs compared to that of rainfall teleconnections (3600 vs. 2500 km) is a likely consequence of the greater heat capacity of seawater inducing long-range autocorrelated patterns in SSTs^31^.

Periods of high temporal connectivity in a TCM correspond to broader spatial scales of synchronization of MHWs. The thick tails of large probabilities observed between 3600 and 6000 km are more pronounced within time windows of significant temporal connectivity for both the historical and the RCP 8.5 scenarios. Similar “bumps” have been observed in a variety of statistical distributions (e.g., earthquakes, epileptic seizures, financial returns, forest fires) and have been named “dragon-kings” to reflect the likely occurrence of extreme events that signal a shift towards a different dynamical regime^18,32,33^. Thus, both TDA and event synchronization concur to indicate the presence of tipping points in the global dynamics of MHWs.

Previous studies have suggested that anthropogenic climate change is projected to increase the probability of MHWs and that the global ocean will reach a nearly permanent MHW state by the end of the twenty-first century^1,13^. When examined through the aggregation of individual MHWs, without consideration of spatial coherence, the transition towards a permanent MHW state of the global ocean appears to be a gradual phenomenon^13^. Instead, by avoiding aggregation and accounting for spatial dynamics, my analysis identifies nonlinearities and thresholds, suggesting that a transition towards a permanent MHW state may occur abruptly rather than gradually in the global ocean. This finding has important implications for how marine life will respond to accelerating warming in the ocean. In particular, abrupt transitions may be more harmful than gradual changes because organisms have less time to adapt and this may lead to increased population collapses and species extinctions^34,35^.

Other studies have considered Earth System Models valuable products to project MHWs to the twenty-first century, albeit with some caveats^1,13^. Specifically, temporal autocorrelation may be stronger in simulated than in real SSTs, which may result in more persistent MHWs. It is known that strong temporal autocorrelation may induce positive covariance effects, potentially overestimating synchronous dynamics in the analysis^19^. Although these effects cannot be excluded, it is important to note that the main transitions observed in this study have occurred within the same networks (historical and RCP 8.5), that is, under the same putative autocorrelation effects of simulated SSTs. In addition, for most of the years when historical and observed data overlap (1982–2005), the corresponding TCMs show the same pattern of non-significant node degree. Finally, the distance distribution of significant synchronization derived for observed MHWs is remarkably similar to the distribution obtained for the most recent historical events. These considerations suggest that the characteristic patterns of temporal connectivity and spatial synchronization of MHWs documented in this study are robust to possible biases originating from the analysis of simulated data.

Research has identified tipping elements in the ocean that may be dangerously close or have already passed a critical threshold at 1.5–2 °C of warming^36^. For example, tropical reefs are projected to collapse worldwide if average SST rises above 2 °C^37^. For other tipping elements of the Earth System, such as the Atlantic meridional overturning circulation (AMOC), evidence suggests that a critical transition may not be expected below 3 °C of warming^38^, although the documented 15% weakening of the AMOC may provide an early warning signal of an approaching tipping point^38,39^. My analysis shows that reducing greenhouse emissions according to the RCP 2.6 scenario would prevent an abrupt shift of the global ocean into an almost persistent ‘blob’, a transition that is projected to occur in the next fifty years under a business-as-usual emission scenario. The RCP 2.6 scenario is compatible with the Paris agreement that aims at limiting global warming well below 2 °C above preindustrial levels. Current national pledges to reduce greenhouse emissions will likely result in at least 3 °C of warming^36^, but this is an unsafe target to safeguard against dangerous shifts in the ocean with potentially dramatic consequences for ecosystems and humanity.

## Methods

### SST data

I used the National Oceanic and Atmospheric Administration (NOAA) daily optimum interpolation SST gridded dataset V2.0 to identify MHWs in the period 1 January 1982 to 31 December 2018^4,28^. The dataset is a blend of observations from satellites, ships and buoys and includes bias adjustment of satellite and ship observations to compensate for platform differences and sensor biases. Remotely sensed SSTs were obtained through the Advanced Very High Resolution Radiometer and interpolated daily onto a 0.25° × 0.25° spatial grid globally. Data were provided by the NOAA/OAR/ESRL PSD, Boulder, Colorado, USA, from their Web site at https://www.esrl.noaa.gov/psd/ accessed in January 2019.

I also obtained data from simulation models implemented in the fifth phase of the Coupled Model Intercomparison Project (CMIP5). I used the first ensemble member r1i1p1 of 12 coupled Earth System Models that allowed the analysis of variation in daily SSTs in all scenarios: CNMR-CM5, GFDL-CM3, GFDL-ESM2G, GFDLESM2M, IPSL-CM5A-LR, IPSL-CM5A-MR, MPI-ESM-LR, MPI-ESM-MR, MIROC5, MIROC-ESM, MRI-CGCM3, MIROC-ESM-CHEM. A climatology (the statistical properties of the timeseries, including the mean, variance, seasonal cycle and quantiles; see section “Identifying marine heatwaves” below for derivation) was obtained from historical simulations over the period 1861–2005 and used to identify MHWs for the historical scenario and for simulated SSTs over the period 2006–2100 following high and low emission scenarios (RCP 8.5 and RCP 2.6, respectively; RCP, representative concentration pathway). All the analyses on simulated data were implemented on a multi-model ensemble obtained by averaging the twelve models, unless otherwise indicated. For comparisons with the simulated SSTs, the satellite 0.25° × 0.25° data were regridded by averaging daily onto a regular 1° × 1° grid. The climatology for the satellite MHWs was derived from the whole observational period (1982–2018).

Whether a fixed climatology is appropriate instead of using shifting baselines to define MHWs is a matter of debate^13,40^. Here, the historical scenario provides a common reference to gauge shifts in the spatiotemporal dynamics of projected MHWs under high (RCP 8.5) and low (RCP 2.6) emission scenarios.

### Identifying marine heatwaves

I identified MHWs from daily observed and simulated SST timeseries within each 1° × 1° cell following Hobday et al.^27^, who define a MHW as an anomalously warm water event with daily SSTs exceeding the seasonally varying 90th percentile (climatological threshold) for at least 5 consecutive days. The climatological mean and threshold were computed for each calendar day within a 11-day window centered on the focal day across all years within the climatological period. The mean and threshold were further smoothed by applying a 31-day moving average. Two events with a break of less than 3 days were considered the same MHW. I then derived characteristic metrics of MHWs, including duration, intensity and frequency and linked them to network properties (see below, Network analysis). Only SST timeseries with less than 10% of missing data were used in the analysis. I used the R package heatwaveR to identify marine heatwaves from SSTs^41^.

### Topological data analysis and the Mapper workflow

Topological Data Analysis (TDA) is a collection of statistical methods based on topology, the field of mathematics that deals with the study of shapes, to find structure in complex datasets^24^. The Mapper algorithm is one tool of TDA that allows reducing high-dimensional data into a combinatorial object that encapsulates the original topological and geometric information of the data, such that points close to each other are more similar than distant points. The combinatorial object, also called a shape graph, is indeed a network with nodes and edges. The statistical properties of the TDA-based Mapper algorithm and how it relates to other non-linear dimensionality reduction techniques have been discussed in Ref.^22^. Here, I briefly summarize the five key steps involved in a Mapper analysis (Fig. 1). The first step of MAPPER consists of collapsing a raster stack of spatiotemporal data of MHWs into a binary 2D matrix where rows are timeframes (days) and columns are 1° × 1° cells arranged sequentially to represent the occurrence of MHWs across the global ocean. The first column of the matrix corresponds to the upper-left pixel of the raster centered at 89.5°N and − 180°W and the subsequent 364 columns represent adjacent pixels within the same latitude. Column 366 is centered at 88.5°N and − 180°W and so on, with the last column of the matrix corresponding to the lower-right pixel at − 89.5°S and 180°E. Although this scheme would result in matrices with 64,800 columns (360 × 180), I used reduced matrices in computations by excluding pixels on land or where missing SST values prevented the identification of MHWs. The final size of the matrices used in the analysis is (rows × columns) 13,514 × 42,365 for observed SSTs and 52,960 × 41,968, 34,675 × 41,074 and 34,675 × 41,482 for simulated SSTs under the historical, RCP 2.6 and RCP 8.5 scenarios, respectively.

The second step of Mapper involves dimensionality reduction or filtering. I used the Uniform Manifold Approximation and Projection dimensionality reduction (UMAP) algorithm to perform nonlinear dimensionality reduction^42^. This algorithm is similar to t-distributed Stochastic Neighbor Embedding (tSNE), which is widely used in machine learning^43^. The advantage of UMAP is that it has superior run time performance compared to tSNE, while retaining the ability to preserve the local structure of the original high-dimensional space after projection into the low-dimensional space.

The third step of Mapper consists of dividing the output range generated by the filtering process into overlapping bins. The number of bins and the amount of overlap are determined by the resolution (*R*) and gain (*G*) parameters, respectively. I used an optimization procedure to objectivity identify the combination of parameters *R* and *G* that best localized timeframes with similar cumulative intensity of MHWs nearby in the network (see “Parameter search and sensitivity analysis”). This procedure selected *R* = 24 and *G* = 45 for observed SSTs and for the RCP 8.5 scenario, *R* = 22 and *G* = 45 for the historical scenario and *R* = 12 and *G* = 25 for the RCP 2.6 scenario.

The fourth step of Mapper consists of partial clustering of timeframes within bins. Although Mapper is flexible and can accommodate different clustering methods and distance functions, I employed single-linkage clustering with Euclidean distance^25^. It is worth noting that this approach does not involve averaging of timeframes within clusters, so the original information is preserved in a compressed representation of the data.

The fifth and final step involves the generation of the network graph from the low dimensional compressed representation of the data. Clusters become nodes in the network and nodes become connected if they share one or more timeframes. I implemented the TDA-based Mapper algorithm using a parallelized version of function mapper2D in the R package TDAmapper^44^.

### Network analysis

I employed two widely used measures of network topology, modularity and node degree, to compare the structure of the four MHW networks. Modularity describes the strength of division of a network into communities—i.e. cohesive groups of nodes that have dense connections among them and that are only sparsely connected with nodes in other groups. High modularity indicates the presence of distinct regimes of spatiotemporal dynamics of MHWs. As a second measure of network structure, I used mean node degree—where the degree of a node is the number of edges that are incident to that node. High mean node degree indicates that many nodes share one or more timeframes and depicts similar spatiotemporal patterns of MHWs within those nodes. In contrast, low node degree indicates the occurrence of many isolated nodes with few timeframes in common and more isolated MHWs. I computed modularity and node degree with functions ‘modularity’ and ‘degree’ in the R package igraph^45^.

To provide significance tests for the observed measures of network topology and to evaluate if they originated simply from non-stationarity properties in the original data, I run two null models based on surrogate data for each of the four networks. Surrogate data can be obtained through the Fourier transform of the original timeseries, shuffling the phases and applying the inverse transform to generate the surrogate series^46^. Phase randomization preserves the power spectrum, autocorrelation function and other linear properties of the data, but not the amplitude distribution. To address this potential drawback, I generated surrogate timeseries via the Theiler's Amplitude Adjusted Fourier Transform (AAFT) using function ‘surrogate’ in the R package fractal, which also preserves the amplitude distribution of the original timeseries^47^. Using this approach, I applied two schemes of randomization—one employing a random sequence for each timeseries and one employing the same sequence for all timeseries. Randomizing using a fixed sequence for all timeseries (constant phase) randomizes the nonlinear properties of the data while preserving linear properties, such as the linear cross-correlation function. The randomization scheme based on random sequences (random phase) also disrupts linear relationships in the data. A significant departure of the observed statistic from the null model under constant phase randomization allows rejecting the null hypothesis that the observed time series is a monotonic nonlinear transformation of a Gaussian process. A significant departure from the null model under random phase allows rejecting also the null hypothesis that the original data come from a linear Gaussian process. To assess significance, 1000 randomizations were performed for each network under each scheme of random and constant phase and a two-tailed test was performed at *α* = 0.025 to account for multiple testing (Bonferroni correction).

To quantify temporal transitions of MHWs, I estimated node degree of the temporal connectivity matrix (TCM) obtained from each network. The degree for each node in the TCM was estimated by counting the number of non-zero edges connected to that node^22^. Temporal fluctuations in node degree were benchmarked against the confidence intervals of the random phase null model, estimated as twice the standard deviation of the null distribution. A Generalized Additive Model (GAM) smooth function was fitted to node degree data to visualize temporal trends. The timing of collapse of node degree for the historical scenario was estimated as the year when the smooth curve intersected the upper confidence limit of the null model. To provide a measure of uncertainty, I obtained analogous estimates of the year of collapse by repeating the whole analysis for each of the twelve ESMs separately and computing the median and the bootstrap standard error (*n* = 1000) of these estimates. A similar analysis was done on the RCP 8.5 data to estimate the year when node degree increased again and diverged significantly from the null distribution. To determine the duration of the different period of connectivity identified in the TCMs, I used the change point algorithm implemented in function cpt.mean of package changepoint^48^.

### Parameter search and sensitivity analysis

To objectively identify parameters *R* and *G* (resolution and gain) as part of the binning process in the Mapper algorithm, I used an optimization procedure that best localized timeframes with similar patterns of MHWs nearby in the network. Localization can be done for any of the properties of MHWs. I used cumulative intensity as the localization criterion since it was a good proxy for other properties of MHWs, such as duration (*r* = 0.88, *p* < 0.0001; averaged across the four datasets) and mean intensity (*r* = 0.31, *p* < 0.0001; averaged across the four datasets). To do this, I repeated the whole TDA-based Mapper analysis over a grid of 120 parameter combinations, by varying *R* from 2 to 30 in steps of 2 and parameter *G* from 5 to 70 in steps of 5. For each analysis I calculated an *F* statistic as the ratio of the variance in MHW cumulative intensity among nodes over the variance within nodes and selected the combination of parameters that maximized *F*.

In addition to the optimization procedure, I also performed a perturbation analysis to evaluate the sensitivity of network properties to selection of parameters *R* and *G.* Sixteen combinations of parameters embracing the values selected by the optimization procedure were examined in this analysis. The statistical properties of the networks under the most extreme combinations of parameters were examined and compared to those of the networks used in the main analysis. Results show that the statistical properties of networks, including node degree and network modularity and the normalized node degree of the temporal connectivity matrix are robust to specific choices of parameters *R* and *G* (Supplementary Figs. 5–8). Therefore, the network properties that underpin the main conclusions of this study are reliable and are largely independent from the specific combination of parameters *R* and *G* used to generate the networks.

### Event synchronization

Event synchronization (ES) is a simple method to quantify the synchrony of events that has been successfully applied for the construction of functional climate networks^18,49,50^. I used the modified version ES that avoids bias due to the consecutive occurrence of events, which can lead to an underestimation of synchronization^18,49^. This bias can be particularly severe for MHWs which, by definition, are periods of extreme warming that last *at least* five consecutive days^27^. To control for this potential bias, I employed the declustering approach used in Ref.^18^, such that events occurring on consecutive days are counted as single events and placed on the first day of occurrence. ES between two timeseries was determined by counting the number of pairs of events that occur closer in time than a local dynamical time delay. Let $e_{1} (m)$ and $e_{2} (n)$ be the time indices for the occurrence of event $m$ and event $n$ in events series $x_{1}$ and $x_{2}$, respectively, and $d_{1,2} = e_{1} (m) - e_{2} (n)$ be the waiting time between the two events. The local time delay is computed as:

$$\tau (m,\;n) = \min \frac{{\{ d_{11} (m,\;m - 1), d_{1,1} (m,\;m + 1),d_{22} (n,\;n - 1), d_{2,2} (n,\;n + 1)\} }}{2}$$

The two events are synchronous if the absolute value of the waiting time $\left| {d_{1,2} } \right|$ is smaller than $\tau (m,\;n)$. To avoid unduly large coincidence intervals, a maximum temporal delay threshold $\tau_{max}$ is set, such that $\left| {d_{1,2} } \right|$ must also be less than $\tau_{max}$. Synchronization between two event series is determined as the sum of occasions that meet the conditions above.

I computed ES between the timeseries for all possible combinations of the 1° × 1° grid cells within time windows corresponding to periods of high or low connectivity in the historical and RCP 8.5 temporal connectivity matrices (TCMs) and for the entire timeseries of contemporary satellite observations. Time windows of ten years were selected in the middle of the first period of significant connectivity and in the middle of the last period of non-significant connectivity in the historical TCM (1891–1900 and 1991–2000, respectively) and the same criterion was used to selected time windows from the RCP 8.5 TCM (2041–2050 and 2081–2090 for periods of non-significant and significant connectivity, respectively). The size of the time window (10 years) was constrained by the relatively short duration of periods of non-significant connectivity in the historical TCM. Alternative analyses compared whole periods of (non)significant connectivity in the historical (1981–1923 vs. 1991–2005) and RCP 8.5 (2006–2070 vs. 2071–2100) TCMs (Supplementary Fig. 9).

To assess the robustness of the results to different choices of the maximum temporal delay threshold, I repeated the analysis for $\tau_{max}$ values of 10 and 30. The statistical significance of ES was determined for each pair of grid cells using surrogate time series. If $l_{i}$ and $l_{j}$ are the number of events of two timeseries, a null-model distribution was obtained under constant phase randomization by computing ES for 2000 pairs of surrogate event series with $l_{i}$ and $l_{j}$ uniformly and randomly distributed events. The 99.5th percentile of the distributions was used as the statistical threshold to assess significance at $\alpha = 0.005$. This stringent criterion was chosen to protect against inflated Type I error rates that may result from the large number of comparisons. Great-circle distances were computed using the Haversine metric to account for spherical embedding.

## Supplementary Information

Supplementary Figures.

## Data Availability

All the data used in this analysis are freely available. CMIP5 data can be accessed at https://esgf-node.llnl.gov/search/cmip5/ and the remotely-sensed satellite SST observations are available at https://psl.noaa.gov/data/gridded/data.noaa.oisst.v2.highres.html. Data to reproduce the results of the paper are deposited on figshare (https://figshare.com/s/ec9061c449031aa2b20e).

## Code availability

The code to reproduce the analyses presented in this study is available from GitHub (https://github.com/bencecc/MHWs.git).
