# Supplementary material for: Complex networks of marine heatwaves reveal abrupt transitions in the global ocean
Source: Sci Rep. 2021 Jan 18;11:1739. doi: 10.1038/s41598-021-81369-3 (PMC7814029; doi:10.1038/s41598-021-81369-3)
Supplement: Supplementary file 1 — Supplementary Figures. [file 41598_2021_81369_MOESM1_ESM.docx]

**Supplementary Information:**

Complex networks of marine heatwaves reveal abrupt transitions in the global ocean

Lisandro Benedetti-Cecchi^1,2,3^

^1^Department of Biology, University of Pisa, Via Derna 1, 56126, Pisa, Italy

^2^Stazione Zoologica Anton Dohrn, Naples 80121 Italy

^3^CoNISMa, Piazzale Flaminio 9, Roma 00196 Italy

e-mail: [lbenedetti@biologia.unipi.it](mailto:lbenedetti@biologia.unipi.it)

voice: +39 050 2211213

ORCID-ID: https://orcid.org/0000-0001-5244-5202


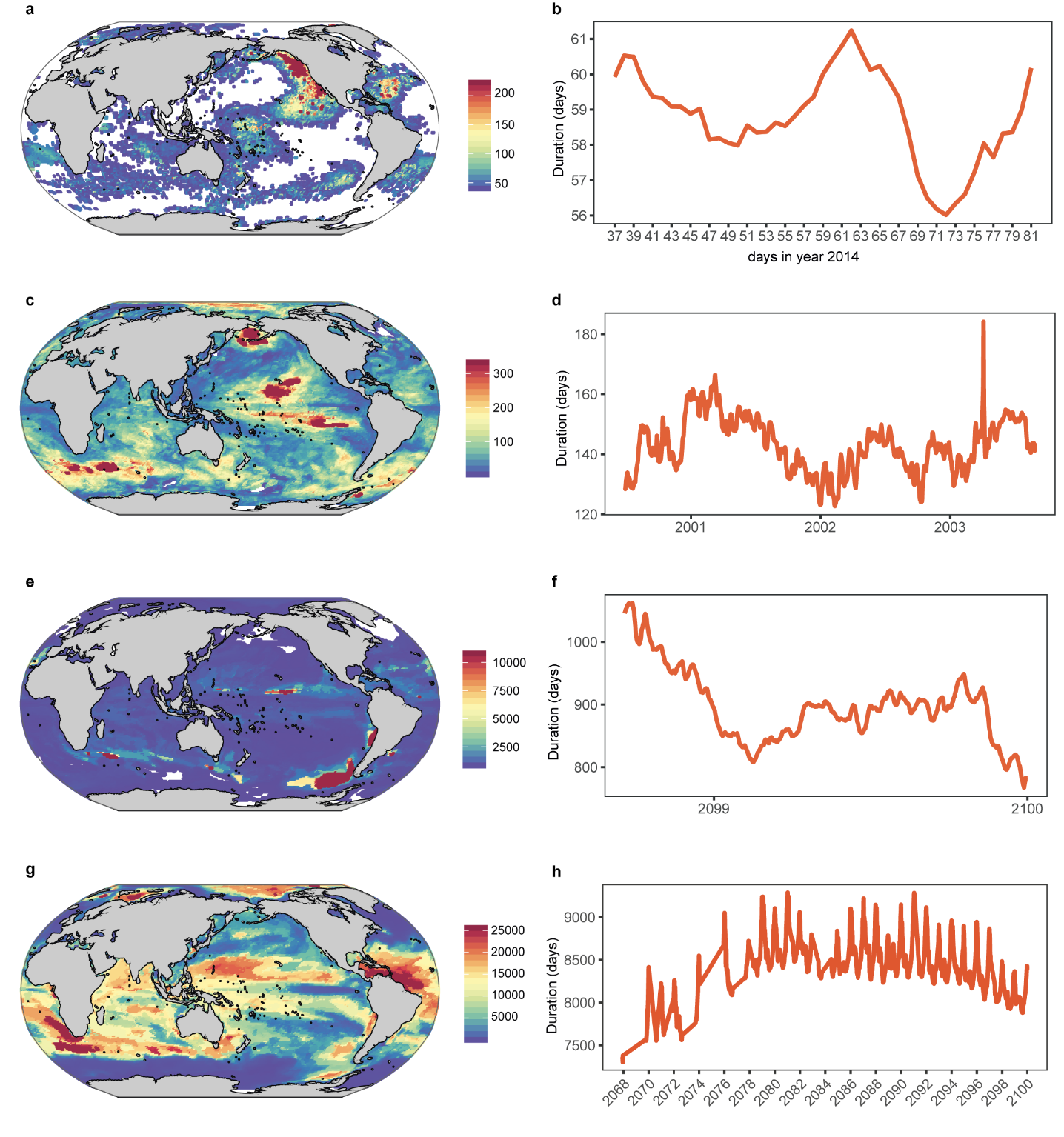


**Supplementary Figure 1**. **Anchoring network topology to the spatiotemporal dynamics of MHWs.** **a, c, e, g,** Maps of mean duration (days) of MHWs corresponding to nodes highlighted in Fig. 2 of main text, including a snapshot of the Pacific northwest MHW named ‘the blob’ from the network of observed MHWs **(a)**, nodes covering the period 2001-2003 from the Historical network **(c)**, nodes including years 2099-2100 from the RCP 2.6 network **(e)** and nodes covering the period 2068-2100 from the RCP 8.5 network showing persistent MHWs pervading the global ocean as a seamless ‘blob’ **(g)**. White areas in the maps have no events for the selected period. Maps were produced in R 4.0.2 (<https://www.R-project.org/>). Corresponding timeseries are shown in the right panels **(b, d, f, h)**. A web application is available to navigate among network nodes, maps and timeseries at: http://calcoloecologia.biologia.unipi.it:3838/MHW_App/.


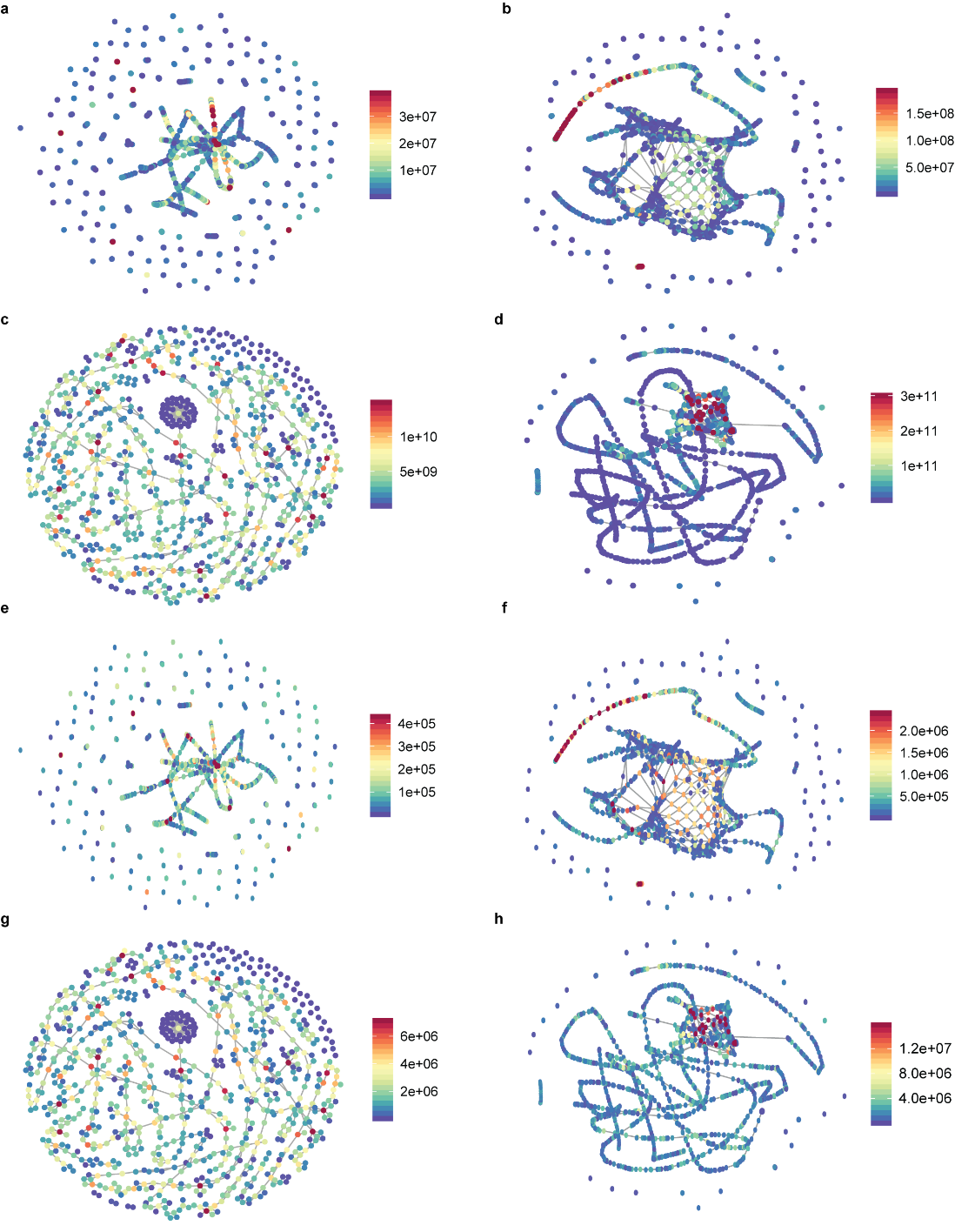


**Supplementary Figure 2**. **Alternative network annotations.** MHW networks generated from remotely sensed SSTs (**a, e)** and from Historical (**b, d**), RCP 2.6 (**c, g**) and RCP 8.5 (**d, h**) simulation scenarios annotated with cumulative intensity (°C x days) (**a-d**) and number (**e-h**) of MHWs. Both measures are cumulated over timeframes within a node and over pixels within a timeframe to generate a single value per node. In agreement to what observed when nodes are colored by duration of MHWs, as in Fig. 2 of main text, events are more frequent and intense towards the end of the simulation period in the historical network (**b, f**) and in the second half of the twenty-first century in the RCP 8.5 network (**d, e**). In contrast, there is no evidence that MHWs become more intense or frequent with time in the RCP 2.6 network (**c, g**). Nodes with the largest cumulative intensity and frequency of events in the observed network are those in the central branch that include the Pacific northwest record-breaking MHW (the blob), as in Fig. 2a in the main text (**a, e**).

**
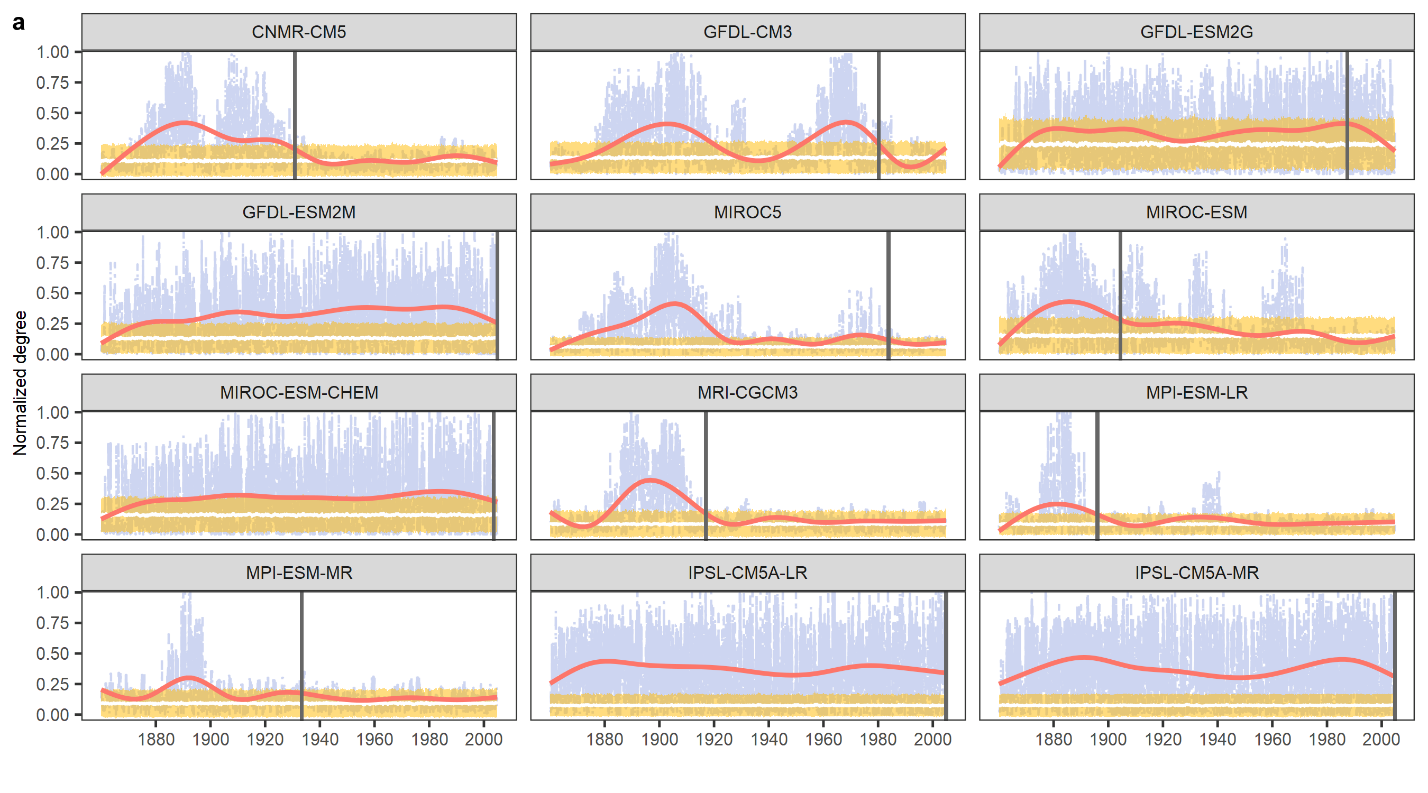
**

**
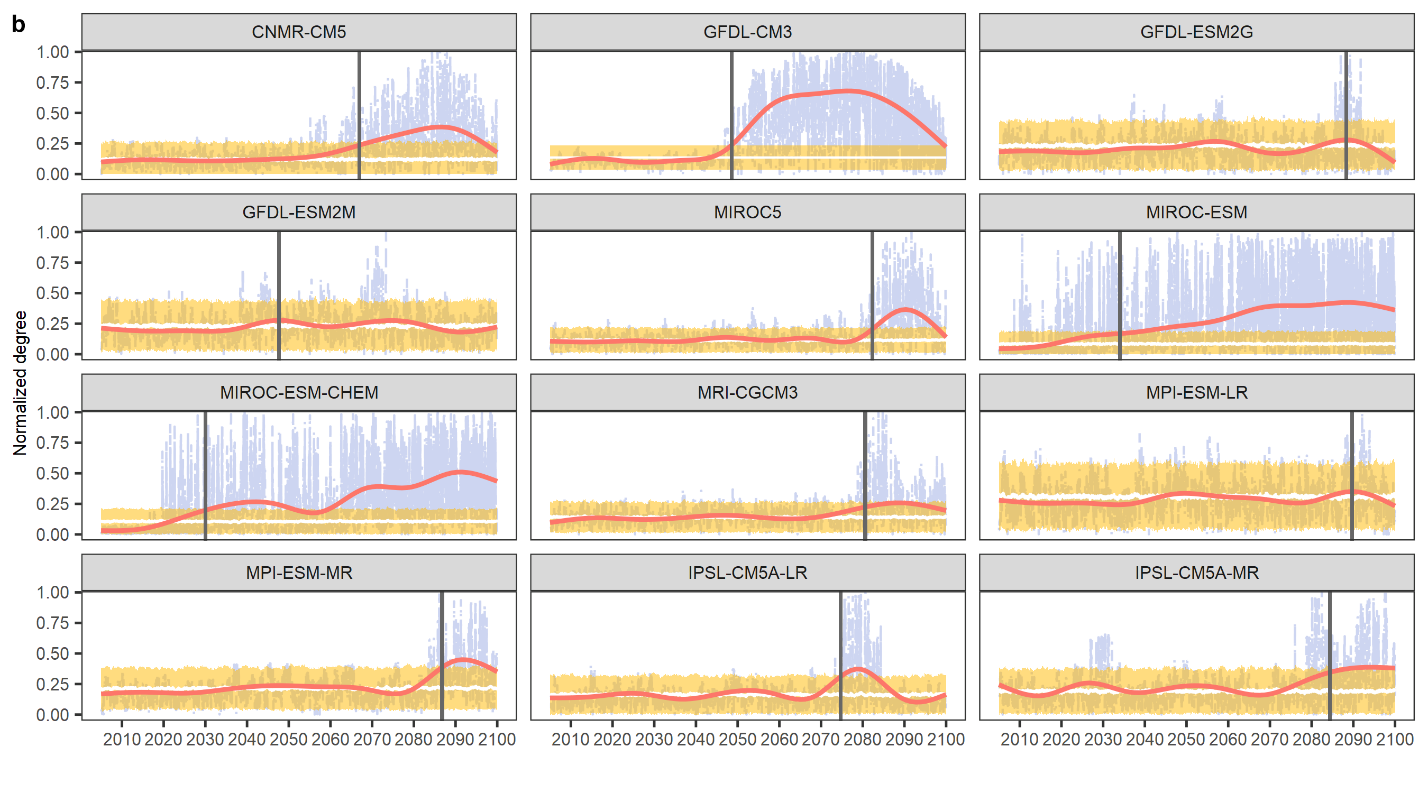
**

**Supplementary Figure 3**. **Node degree of temporal connectivity matrices (TCMs) from individual Earth System models.** **a**, Historical and **b**, RCP 8.5 scenario. Yellow bands are confidence intervals of random phase null models (with average trend in white); vertical gray lines show years of transition determined from the intersection of GAM fits (in red) with the upper confidence limit of null models. The transition year for models where the GAM fists does not intersect the confidence interval corresponds to the last day of the simulation for historical models (IPSL-CMSA-LR and IPSL-CMSA-MR) or to the maximum value of the GAM curve for RCP 8.5 models (GFDL-ESM2G, GFDL-ESM2M, MPI-ESM—LR).


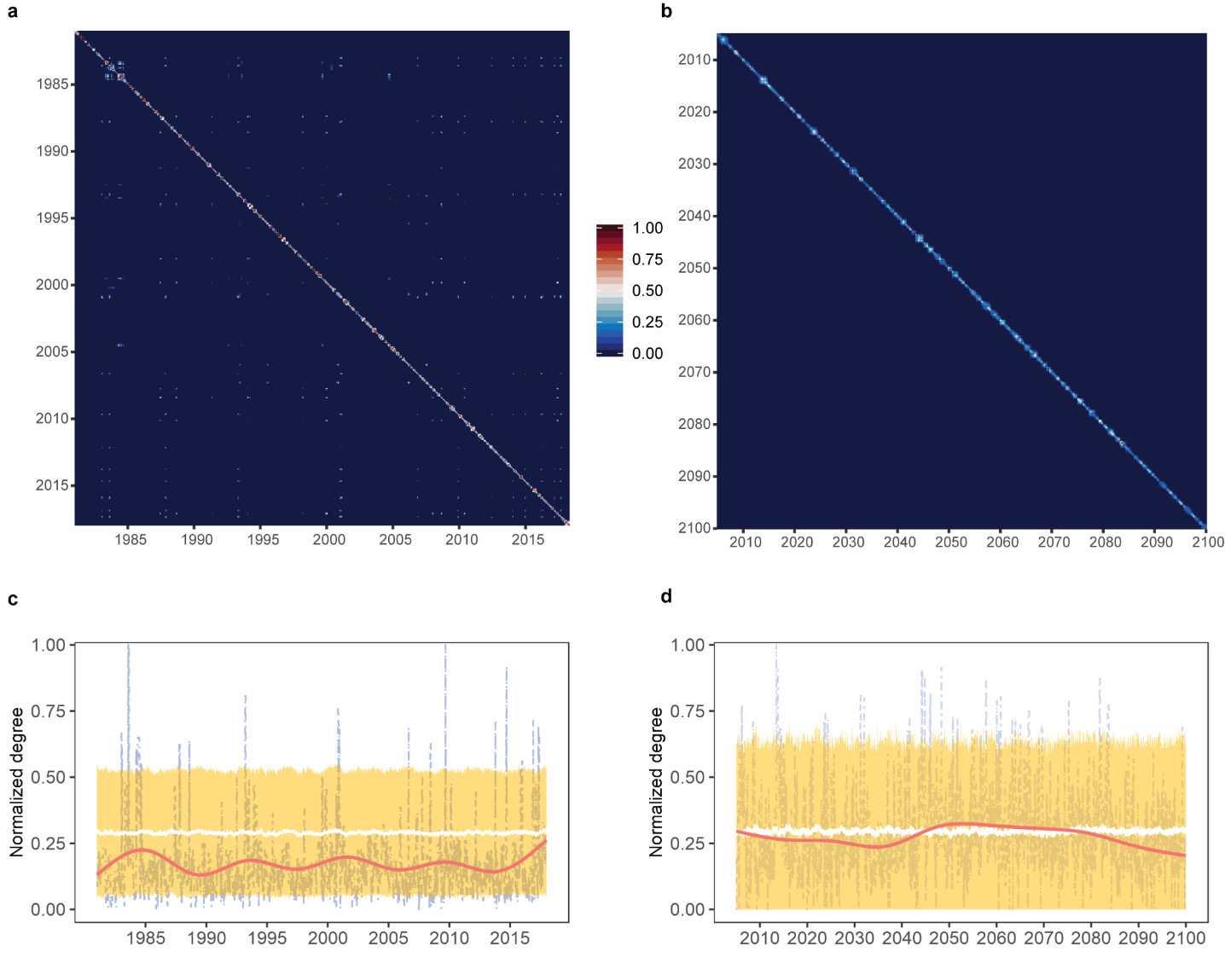


**Supplementary Figure 4**. **Temporal transitions of global MHW dynamics.** Panels show temporal connectivity matrices (TCMs) originating from (**a),** observed and (**b),** RCP 2.6 networks. A TCM shows the similarity of each timeframe with all other timeframes. Similarity is negligible compared to the TCMs originated from Historical and RCP 8.5 networks shown in Fig. 4 of main text. Temporal trajectories of node degree are shown for observed (**c**) and RCP 2.6 (**d**) TCMs. Red lines in **c, d,** are Generalized Additive Model (GAM) fits to node degree data; yellow bands are confidence intervals of random phase null models (with average trend in white). Average node degree is not statistically distinguishable from null models.


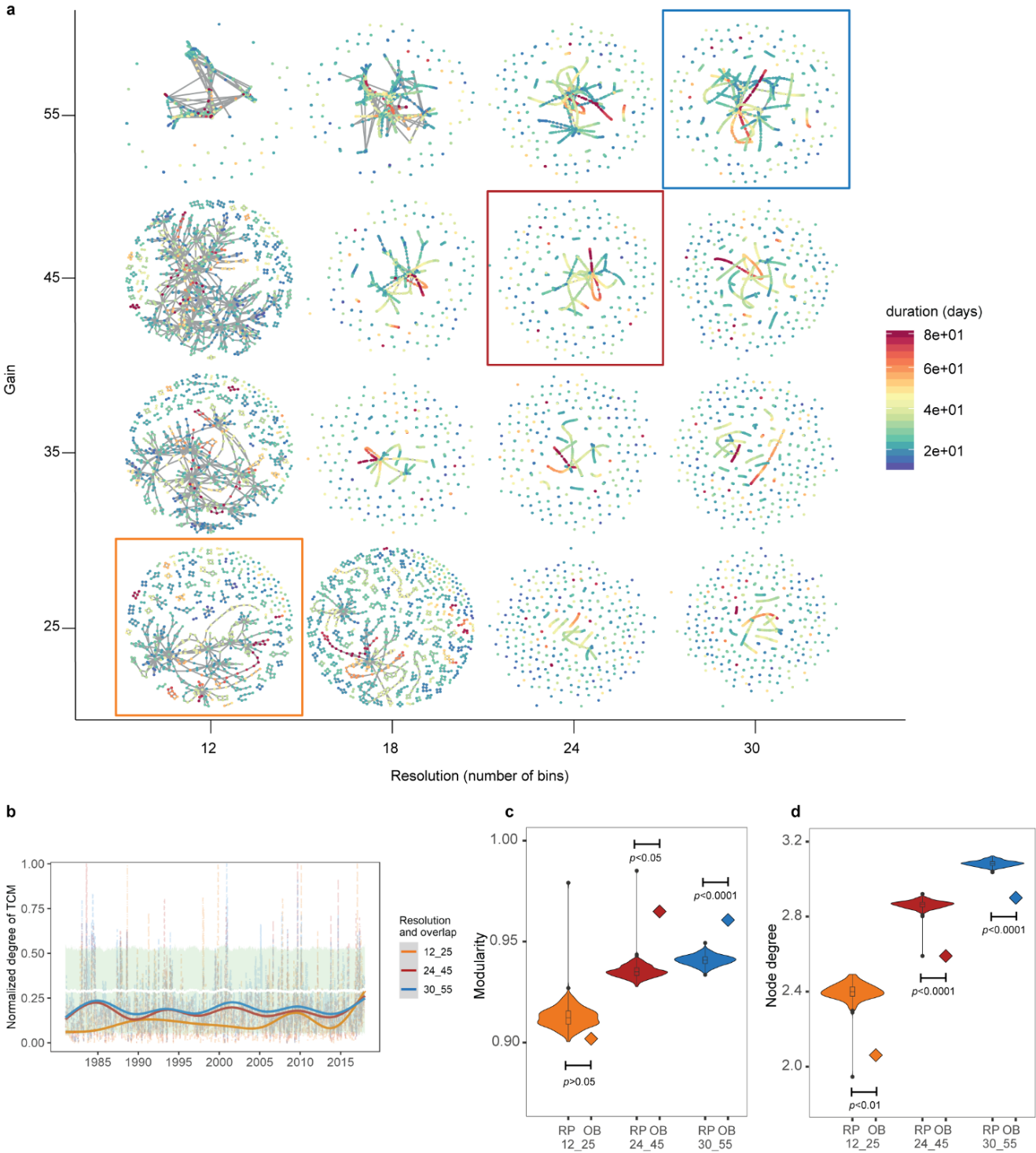


**Supplementary Figure 5. Effects of perturbing TDA-Mapper parameters on networks of observed MHWs.** **a,** comparison of 16 combinations of Resolution and Gain parameters. The red frame indicates the network selected by the optimization procedure. The orange and blue frames indicate the alternative networks used to assess sensitivity in statistical properties of networks. **b,** Node degree of the temporal connectivity matrix (TCM) for the three networks. In all cases node degree is not distinguishable from the random phase null model derived from the selected network (see also Supplementary Fig. 4c). **c,** Modularity of the three networks. The graph shows violin and box plots originating from the random phase null model (RP) and the observed value of the statistic (OB). Observed modularity for the network with the lowest values of resolution and gain (12 and 25, respectively) does not differ statistically from the null model. In contrast, observed modularity is significantly larger than expected in the other networks. **d,** Node degree of the three networks. In all instances observed node degree (OB) is significantly lower than in the corresponding null model (RP). Overall, this analysis shows that the network of observed MHWs has low spatiotemporal connectivity and this outcome does not depend on the specific combination of the resolution and gain parameters used to generate the network.

**
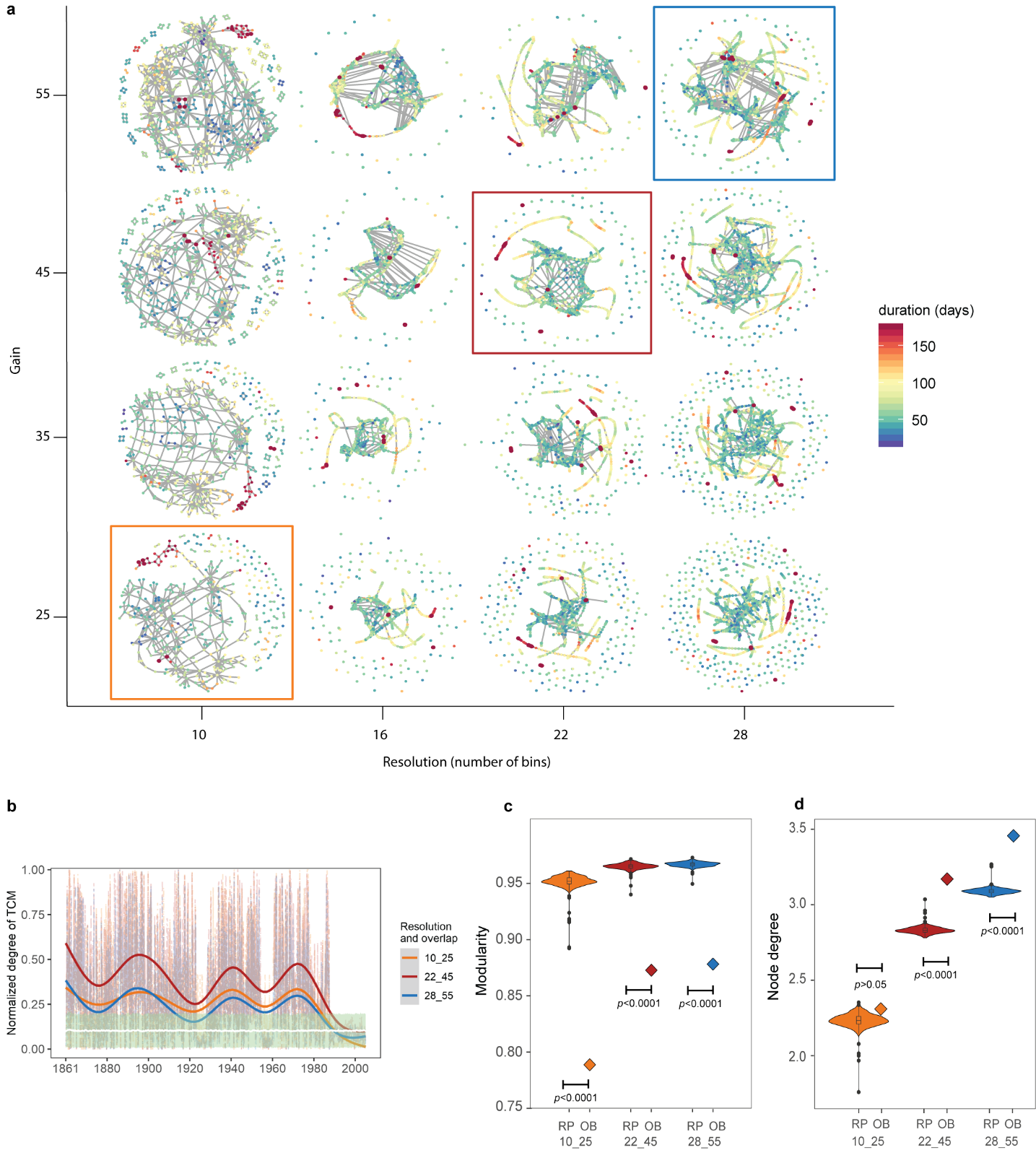
**

**Supplementary Figure 6. Effects of perturbing TDA-Mapper parameters on networks of historical MHWs.** **a,** comparison of 16 combinations of Resolution and Gain parameters. The red frame indicates the network selected by the optimization procedure. The orange and blue frames indicate the alternative networks used to assess sensitivity in statistical properties of networks. **b,** Node degree of the temporal connectivity matrix (TCM) for the three networks. In all instances node degree raises above the upper confidence limit of the random phase null model of the selected network. **c,** Modularity of the three networks. The graph shows violin and box plots derived from the random phase null model (RP) and the observed value of the statistic (OB). In all cases observed modularity is significantly lower than expected from null models. **d,** Node degree of the three networks. Observed degree for the network with the lowest values of resolution and gain (10 and 25, respectively) does not differ statistically from the null model. In contrast, node degree is significantly larger than expected in the other networks. Overall, this analysis shows that the declining temporal trend of network connectivity and its collapse in the late ‘80s is a robust result that does not depend on the specific combination of the resolution and gain parameters used to generate the network.


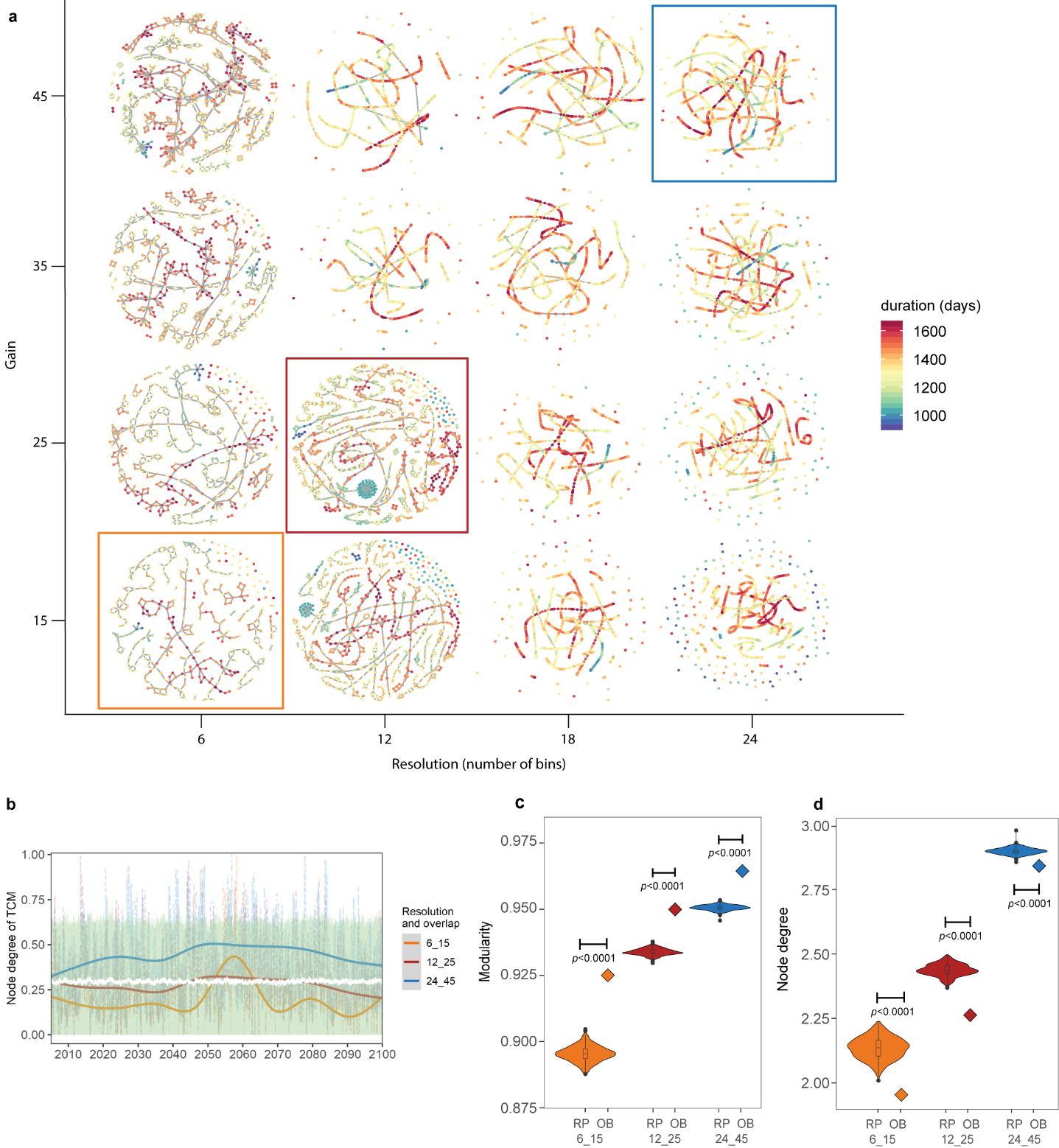


**Supplementary Figure 7. Effects of perturbing TDA-Mapper parameters on networks of RCP 2.6 MHWs.** **a,** comparison of 16 combinations of Resolution and Gain parameters. The red frame indicates the network selected by the optimization procedure. The orange and blue frames indicate the alternative networks used to assess sensitivity in statistical properties of networks. **b,** Node degree of the temporal connectivity matrix (TCM) for the three networks. In all cases node degree is not distinguishable from the random phase null model derived from the selected network. **c,** Modularity of the three networks. The graph shows violin and box plots derived from the random phase null model (RP) and the observed value of the statistic (OB). In all cases observed modularity is significantly larger than expected from null models. **d,** Node degree of the three networks. Observed node degree (OB) is always significantly lower than in null models (RP). Overall, this analysis shows that the network of observed MHWs has low spatiotemporal connectivity and this outcome does not depend on the specific combination of the resolution and gain parameters used to generate the network.


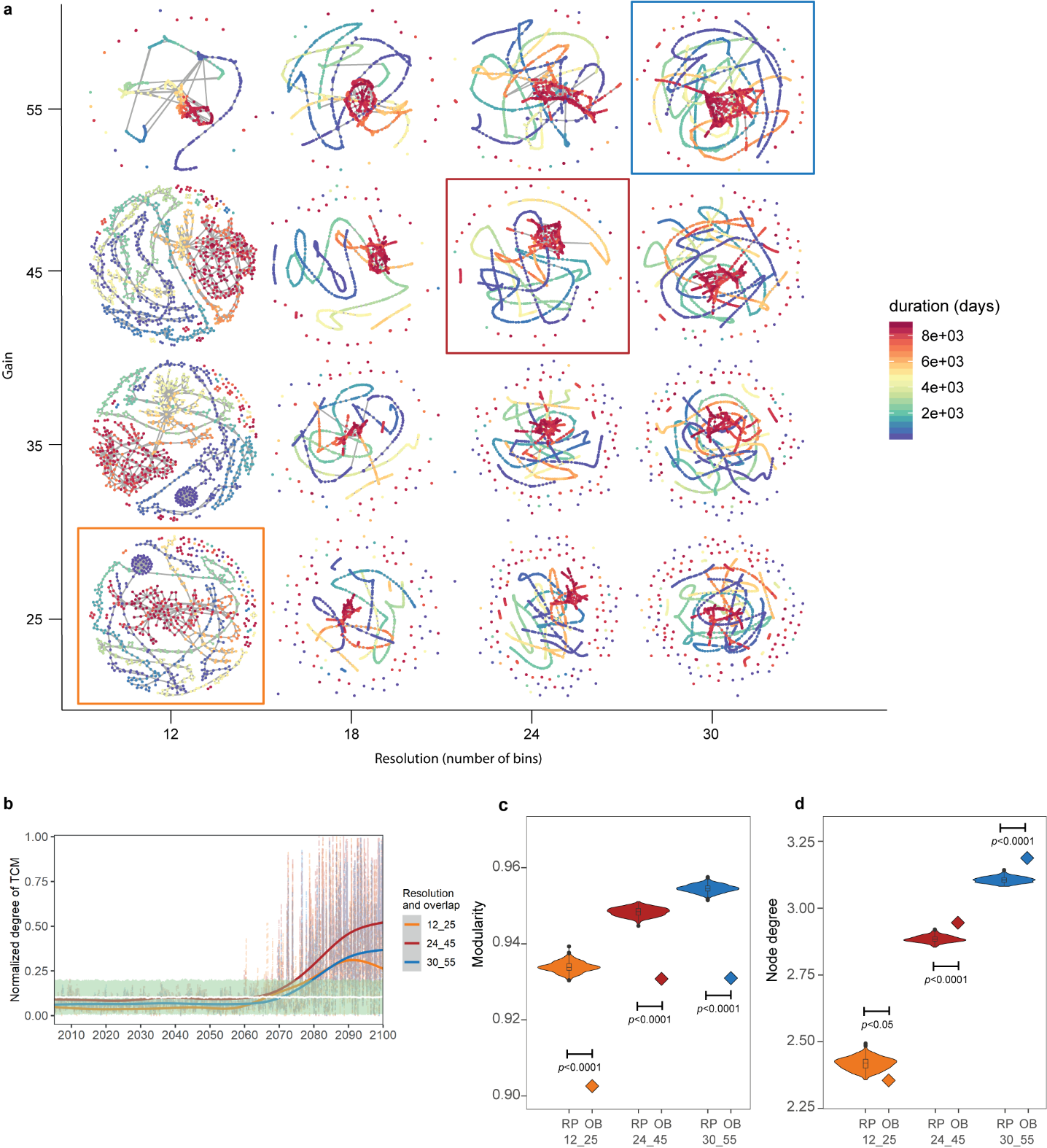


**Supplementary Figure 8. Effects of perturbing TDA-Mapper parameters on networks of RCP 8.5 MHWs. a,** comparison of 16 combinations of Resolution and Gain parameters. The red frame indicates the network selected by the optimization procedure. The orange and blue frames indicate the alternative networks used to assess sensitivity in statistical properties of networks. **b,** Node degree of the temporal connectivity matrix (TCM) for the three networks. In all cases node degree raises above the upper confidence limit of the random phase null model of the selected network in the second half of the twenty-first century. **c,** Modularity of the three networks. The graph shows violin and box plots originating from the random phase null model (RP) and the observed value of the statistic (OB). Observed node degree (OB) is always significantly lower than in null models (RP). **d,** Node degree of the three networks. Observed degree for the network with the lowest values of resolution and gain (12 and 25, respectively) is significantly lower than in the corresponding null model. In contrast, observed node degree is significantly larger than expected in the other networks. Overall, this analysis shows that a significant increase in spatiotemporal connectivity of MHWs can be expected in the next decades under a wide range of combinations of the resolution and gain parameters used to generate the networks.


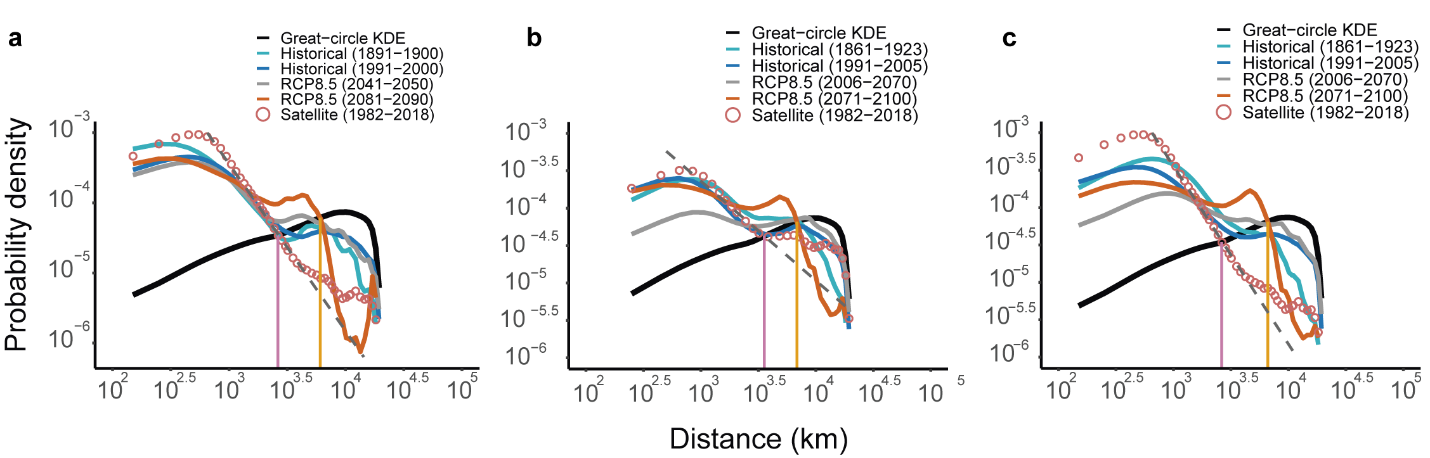


**Supplementary Figure 9. Distance distributions of MHW synchronization for different time windows and values of** $\boldsymbol{\tau}_{\boldsymbol{max}}$**. a,** as in Fig. 5 of main text, but with $\tau_{max}=30$. The pink vertical line marks the intersection between the power-law of the satellite-derived distribution and the distribution of all possible great-circle distances (at about 2,600 km). The yellow vertical line shows extended spatial scales of synchronization at about 6,000 km for the thick-tailed distribution (RCP 8.5 in the period 2081-2090). **b,** distance distributions for the entire period of significant connectivity observed for the first decades of the historical scenario (1861-1923) and for the latest period of non-significant connectivity (1991-2005). Similarly, distance distributions have been obtained for the whole period of non-significant connectivity of the RCP 8.5 scenario (2006-2070) and for the latest period after a shift to significant connectivity (2071-2100). The pink vertical line marks a scale-break in the power law of the satellite-derived distribution at about 3,600 km as in the analysis reported in main text. The yellow vertical line shows extended spatial scales of synchronization at about 6,800 km for the thick-tailed distributions of historical and RCP 8.5 distances. Analysis based on $\tau_{max}=10.$ **c,** as in **(b)**, but with $\tau_{max}=30$. Here, the pink and yellow vertical lines mark distances at about 2,600 and 8,600 km. The results of these alternative analyses are qualitatively similar to those presented in Fig. 5 of main text, although the differences in large-scale synchronization between the historical and the RCP 8.5 scenarios increase and the break-point in the satellite-derived distribution is less obvious with $\tau_{max}=30$.
